# Syntaxin-6 promotes the progression of hepatocellular carcinoma and alters its sensitivity to chemotherapies by activating the USF2/LC3B axis

**DOI:** 10.7150/ijbs.86636

**Published:** 2023-07-31

**Authors:** Lianer Zhou, Zhenyu Wang, Xiaoxia Chen, Xianxian Li, Chao Ge, Xuejie Min, Fangyu Zhao, Taoyang Chen, Jinjun Li

**Affiliations:** 1 State Key Laboratory of Systems Medicine for Cancer, Renji Hospital, Shanghai Jiao Tong University School of Medicine, Shanghai 200032, China.; 2 Department of Oncology, Shanghai General Hospital, Shanghai Jiaotong University School of Medicine, Shanghai 200080, China.; 3 School of Life Science and Technology, Shanghai Tech University, Shanghai 201210, China.; 4 Qi Dong Liver Cancer Institute, Qi Dong 226200, China.

**Keywords:** STX6, hepatocellular carcinoma, metastasis, proliferation, drug resistance

## Abstract

Syntaxin-6 (STX6), a protein of the syntaxin family, is located in the trans-Golgi network and is involved in a variety of intracellular membrane transport events. STX6 is overexpressed in different human malignant tumors. However, little is known about its exact function and molecular mechanism in hepatocellular carcinoma (HCC). In this study, we found that the expression of STX6 was significantly increased in HCC tissues and was associated with poor survival. Gain- and loss-of-function experiments showed that STX6 promotes cell proliferation and metastasis of HCC cells both in vitro and in vivo. Mechanistically, STX6 was negatively regulated by the upstream stimulatory factor 2 (USF2). In addition, STX6 facilitates the association of autophagosomes with lysosomes. Importantly, we demonstrated that STX6 overexpression, despite enhanced resistance to lenvatinib, sensitizes HCC cells to the autophagy activator rapamycin. This study revealed that, under the control of USF2, STX6 accelerates the degradation of microtubule-associated protein 1 light chain 3 beta (LC3) by promoting autophagic flux, ultimately promoting HCC progression. Collectively, we suggest that the USF2-STX6-LC3B axis is a potential therapeutic target in liver cancer.

## Introduction

Hepatocellular carcinoma (HCC) accounts for more than 80% of all primary liver cancers and is the fourth leading cause of cancer-related deaths worldwide^1^. The five-year survival rate of HCC is only 18%, and its mortality rate is second only to that of pancreatic cancer^2^. Despite recent advances in chemoradiotherapy and surgery, current treatments for HCC can't effectively improve the prognosis of patients. Moreover, proliferative HCCs are characterized by the activation of signaling pathways involved in cell proliferation^3^. Therefore, it is urgent to identify an effective molecular pathway target for the treatment of HCC.

Autophagy is a crucial cellular degradation program that removes excess products from cells and maintains homeostasis. It involves two steps: the formation of double-membrane autophagosomes and the fusion of mature autophagosomes with lysosomes 4. Autophagy plays a dual role in tumor cell development. Pietrocola et al. reported that autophagy-inducing caloric restriction mimetics improve the antitumor efficacy of chemotherapy *in vivo*^5^. Another study proposed that inhibition of autophagy induces changes in the tumor microenvironment, ultimately leading to Treg-mediated inhibition of anticancer immunosurveillance^6^. In HCC, the autophagy-mediated ubiquitin-proteasome system inhibits the phosphatase and tensin homolog system to activate the AKT-PI3K-mTOR pathway. LC3 is a widely used autophagy marker that marks autophagic structures at different stages of biogenesis. Recent advances have shown that an autophagy-independent function of lipidated LC3 can activate transcription factor EB (TFEB) regulating autophagy and lysosomal biogenesis during lysosomal damage^7^. Therefore, the situation of autophagy in HCC can be explored by observing the changes in LC3 molecules.

Syntaxin-6 (STX6) is a soluble *N*-ethylmaleimide-sensitive factor attachment protein receptor (SNARE) protein. SNARE proteins are classified, according to their central residues in the SNARE motifs, into R- (from arginine) and Q- (glutamine) SNAREs^8^. SNARE proteins participate in almost all intracellular membrane trafficking events, including endosomal and phagosomal trafficking^9^. STX6 is critical for anterograde and retrograde endosomal trafficking in the Golgi, and knockout of STX6 results disrupts the Golgi ribbon structure^10^. Anterograde trafficking from the Golgi apparatus to the endosome is regulated by the interaction of STX6 with members of the mammalian E3 ubiquitin ligase family. The recruitment of STX6 helps phagosomes clear intracellular pathogens such as Mycobacterium tuberculosis^11^, ^12^. Moreover, the overall expression of STX6 is elevated in different human tumors^13^. However, little is known about the role of STX6 in HCC development.

In this study, we analyzed the expression level and clinical significance of STX6 in patients with HCC and studied the molecular mechanisms underlying its role. Mechanistically, STX6 promoted the autophagic flux in HCC cells, ultimately promoting HCC progression. This study reveals that STX6 is a potential target for the treatment of HCC.

## Materials and methods

### Cell lines and cell culture

The human primary HCC cell line MHCC-97H was provided by the Liver Cancer Institute of Zhongshan Hospital, Fudan University (Shanghai, China). Huh7 was purchased from the Riken Cell Bank (Tokyo, Japan). Li-7 cells were obtained from the Cell Bank of the Institute of Biochemistry and Cell Biology, China Academy of Sciences (Shanghai, China). The Hep3B and HEK-293T cell lines were provided from the American Type Culture Collection (Manassas, VA, USA) ATCC. Cells were all cultured in Dulbecco's modified Eagle's medium (DMEM, Gibco, 11965092) containing 10% fetal bovine serum (FBS) (Gibco, 10100147C) 100 μg/mL streptomycin, and 100 U/mL penicillin (Gibco, 15070063) maintained at 37℃ with 5% CO_2_.

### Clinical tissue samples

Clinical tissue samples were supplied by the Guangxi Cancer Institute (Nanning, China), Zhejiang University (Hangzhou, China), and the Qidong Liver Cancer Institute (Qidong, China). This study was approved by the Research Ethics Committee of Renji Hospital, Shanghai Jiao Tong University School of Medicine in accordance with the Declaration of Helsinki. Written informed consent was obtained from all participants prior to inclusion in this study.

### Plasmid construction and transfection

The plasmids coding sequences for human STX6(NM_005819.6) and its shRNA lentiviral plasmids were supplied by GeneCopoeia (Guangzhou, China). The USF2 lentiviral plasmids were obtained from Genechem (Shanghai, China). psPAX2 and pMD2.G plasmids were purchased from Addgene (USA). And the shRNA target sequences are listed in Table S1. Lipofectamine 2000 reagent (Invitrogen, 11668030) and overexpressing or interfering plasmid-mixed psPAX2 and pMD2.G plasmids were transfected into 293T cells. HCC cells were infected with 1×10^6^ recombinant lentivirus-transducing units in the presence of 6 μg/mL polybrene (Sigma-Aldrich, 107689).

### RNA isolation and qRT-PCR

RNA isolation and QRT PCR were performed as previously reported procedures^14^. Primer sequences are listed in Tables S1 and S2.

### Western blot analysis

Western blotting was conducted as previously reported^14^. Detailed information on the antibodies used is shown in Table S3.

### In vitro cell behavior assays

Cell proliferation, colony formation assays, and Transwell assays were performed as previously reported^14^.

### Immunofluorescence studies

Cells were cultured in Earle's balanced salt solution (Beyotime, C0213-500) for more than 6 h. The cells were then washed thrice with PBS in a confocal dish (NEST, 801001) and fixed with 4% paraformaldehyde for 15 min. Then DAPI (Invitrogen, D1306) in a blocking solution for 30 min. Images were taken using a Zeiss fluorescence microscope (Axio Vert.A1; Carl Zeiss AG, Jena, Germany).

### Drug sensitivity assays

For short-term drug toxicity experiments, sirolimus (Sigma-Aldrich, S-015) was prepared as a solution at different concentrations and added sequentially to 96-well plates. The same number of cells was inoculated into the wells, and cell viability was detected using the CCK8 assay (Bimake, B34304) after culturing for 72h.

### Flow cytometry analysis

The cells were seeded into 6-well plates. After adherence, cells were incubated with drugs for 24 h. The cells were then stained with eBioscienceTM Annexin V Apoptosis Detection Kit (Thermo Fisher Scientific, 88-8007). Analysis was performed using flow cytometry within 4 h.

### Chromatin immunoprecipitation assay

ChIP assays were performed using a ChIP Assay Kit (Millipore, MAGNA0017) according to the manufacturer's protocol. The cell lysates were sonicated to shear the DNA to 500-1,000 bp fragments. The supernatant was mixed with a mouse anti-USF2 antibody, mouse IgG, and magnetic beads with rotation at 4 °C overnight. Subsequently, magnetic beads were collected, washed, and purified in multiple steps. Purified DNA samples were extracted for subsequent qRT-PCR and agarose electrophoresis. Primer sequences are listed in Table S3.

### Coimmunoprecipitation assay

Proteins were extracted from HCC cells using RIPA reagent mixed with protease inhibitors on ice and centrifuged at 12,000 ×*g* for 10 min. Cell lysates were divided into three groups and incubated with antibodies, negative control IgG, and protein A/G agarose beads, respectively, overnight with rotation at 4℃. The beads were washed thrice and collected for western blot analysis. The antibodies used are listed in Supplementary Table S3.

### Dual-luciferase reporter assay

Recombinant plasmids with the normal and truncated promoter regions of STX6 were purchased from IGEbio (Guangzhou, China). HCC-LY10 and PLC/PRF/5 cells were transiently transfected with their corresponding reporter plasmids. Luciferase activity was measured in the cells following the manufacturer's instructions (Promega, E1980).

### Immunohistochemical (IHC) staining

Paraffin-embedded tissue microarray (TMA) sections were deparaffinized with xylene, then rehydrated in graded alcohol. Subsequently, following routine IHC procedures, the sections were incubated with STX6 antibody at 4°C overnight. The cells were finally scored according to their staining intensity. Staining intensity was defined as 0, 1, and 2, indicating negative, weak, and strong positive respectively.

### In vivo tumor formation assays

For *in vivo* liver orthotopic tumor transplantation experiments, randomized six-week-old male BALB/c mice were raised in the Laboratory of Experimental Pathology (n=10). MHCC-97H cells (2×10^6^) suspended in 50 μL of FBS-free DMEM containing Matrigel (BD Biosciences, 354230) were injected into the left hepatic lobe.

For *in vivo* subcutaneous tumor xenografting, Huh7 cells (2×10^6^) were resuspended in 200 μL FBS-free DMEM and inoculated subcutaneously into one flank of each male nude mouse (n=10). Several weeks later, all mice were sacrificed, and the length, short diameters, and weights of tumors were measured. All mouse experiments were approved and conducted according to the protocol of the Institutional Animal Care and Use Committee of the Renji Hospital.

For tail vein injection of tumor cells animal model, each BALB/c nude mouse was injected via the tail vein with 2 × 10^6^ Li-7 cells transfected with the STX6 overexpression vector or the blank plasmid. After eight weeks, nude mice were sacrificed. Lung tissues were removed and imaged, and the number of nodules on the surface of the lung was recorded to assess tumor metastasis. Lung tissues were then subjected to gradient dehydration, sectioned, embedded in paraffin, and stained with H&E for histological examination.

### Statistical analysis

All data were analyzed using GraphPad Prism software (version 7.0), ImageJ software, and SPSS16.0 software. For statistical comparisons of data, a two-tailed Student's *t*-test was used for two-group comparisons or one-way analysis of variance ANOVA. The correlation coefficient was determined using Pearson correlation analysis. All data are shown as mean ± SD. Statistical significance was set at *p* < 0.05 (* *p* < 0.05; ** *p* < 0.01).

## Results

### STX6 is overexpressed and correlates with poor prognosis in HCC

**Figure 1:**
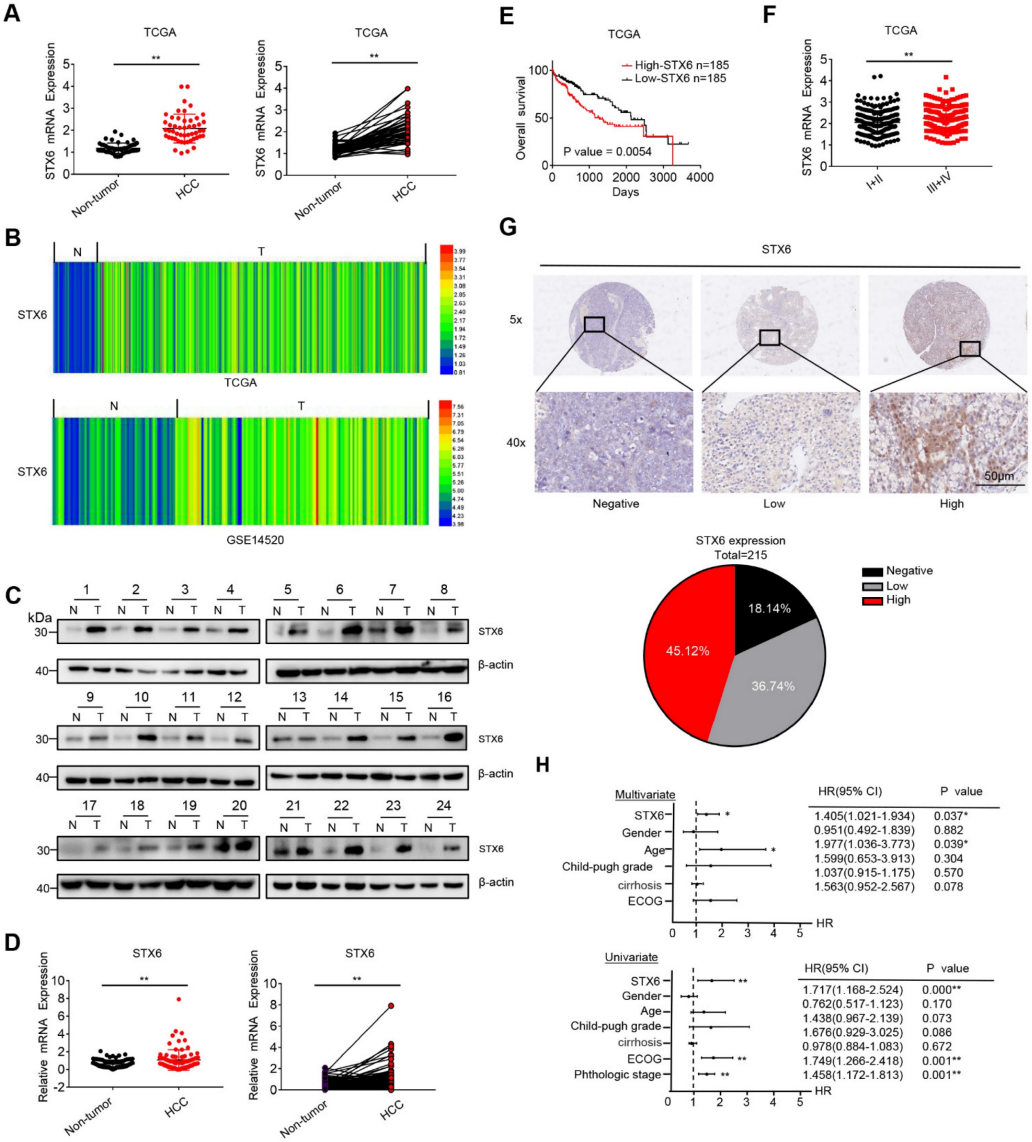
STX6 is upregulated in HCC and associated with poor prognosis. (**A and B**) STX6 expression in HCC and the corresponding nontumorous liver tissues in the TCGA dataset and GSE14520. (**C**) Western blot of STX6 expression in 24 paired human HCC tissues and the matched noncancerous liver tissues. (**D**) STX6 mRNA expression in 88 paired human HCC tissues and the matched noncancerous liver tissues in our lab were evaluated by qRT-PC. (**E**) Kaplan-Meier analysis of the correlation between STX6 expression and overall survival of patients with HCC in the TCGA dataset (n=370). (**F**) Expression of STX6 in human primary HCC tissues with early (I-II, n=243) and advanced pathological stages (III-IV, n=167) in the TCGA database. The data are presented as mean ± SD. * *p* < 0.05; ** *p* < 0.01; by two-tailed Student's *t*-test. (**G**) Representative images of IHC staining of STX6 protein expression in human primary HCC tissues. The statistical analysis is shown in the lower pie chart. Original magnification: 5× (upper panel) and 40× (lower panel). (**H**) Multivariate and Univariate Cox proportional hazards analyses of the association between STX6 and the overall survival of in patients with HCC. *p < 0.05; **p < 0.01. HCC, hepatocellular carcinoma; STX6, syntaxin-6; OS, overall survival; HR, hazard ratio; TCGA, The Cancer Genome Atlas; GEO, Gene Expression Omnibus.

We first analyzed the expression level of STX6 in the tumors and noncancerous tissues of patients in The Cancer Genome Atlas (TCGA), HCCDB, and Gene Expression Omnibus (GEO) databases. The results revealed that the expression of STX6 was upregulated in HCC tissues compared to adjacent tissues (Fig. 1A, B, and S1A). The expression level of STX6 in HCC was also higher than that in adjacent tissues in the UALCAN database (Fig.S1C). To validate this result, we performed quantitative real-time PCR assays (RT-PCR) and western blot assays using tumor and matched noncancerous tissues obtained from patients with HCC to detect the expression of STX6 (Fig. 1C, D, and S1B). Consistent with the databases, STX6 was overexpressed in HCC tissues compared to noncancerous tissues.

Furthermore, we investigated the clinical significance of STX6 expression in patients with HCC using Kaplan-Meier analysis and found that patients with HCC with high STX6 expression had a poor prognosis (overall survival [OS]; *p* = 0.0054), the results of the UALCAN database are consistent with our analysis of the TCGA database (Fig. S1D). And STX6 expression was positively correlated with histological grade (Fig. 1E, F). In addition, the IHC analysis results of clinical HCC samples showed that the expression of STX6 was in direct proportion to the histological stage of the HCC patients, which was consistent with the analysis results of the TCGA database (Fig. 1G and Table S4). Moreover, univariate and multivariate Cox proportional hazard analyses suggested that high STX6 expression was associated with poor survival in patients with HCC (Fig. 1H). These results indicated that STX6 was overexpressed in HCC and is a potential prognostic factor for patients with HCC.

### STX6 promotes HCC cell growth and tumorigenicity in vitro and in vivo

**Figure 2:**
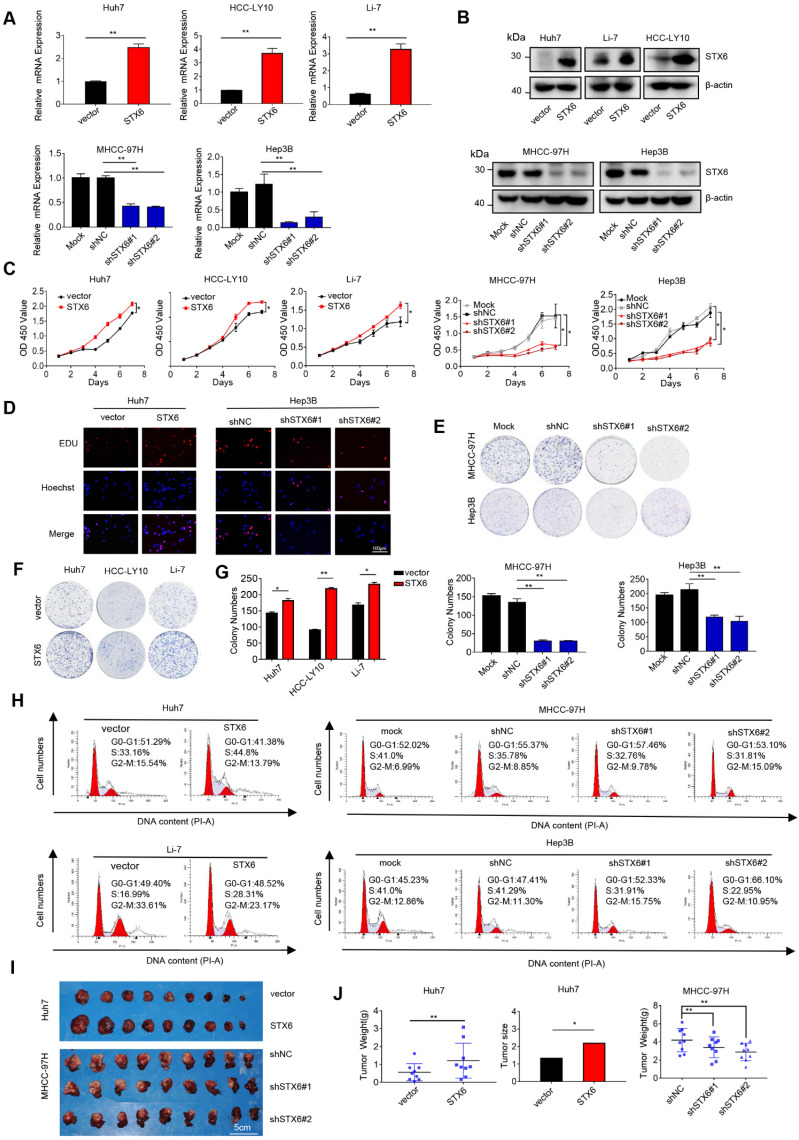
STX6 promotes HCC cell growth. (**A and B**) STX6 mRNA and protein level detection in STX6-overexpressing and STX6-deficient HCC cells. (**C**) Cell Counting Kit 8 assay of the proliferation of HCC cells with STX6 overexpression or knockdown. (**D**) Representative images of the EdU labeling assays of HCC cells with STX6 overexpression or knockdown. Scale bar: 100 µm. (**E-G**) Colony formation assay of HCC cells with STX6 overexpression or knockdown. (**H**) Flow cytometry analysis of the cell cycle MHCC-97H and Hep3B cells with STX6 knockdown and Huh7 and Li-7 cells with STX6 overexpression. (**I**) Tumor tissues inoculated from xenograft-bearing animals with STX6-overexpressing Huh7 and STX6-deficient MHCC-97H cell lines. (**J**) Quantitative data analysis of tumor weight and volume. Data for the *in vitro* experiments are the means ± SDs and are representative of three independent experiments. *p < 0.05, **p < 0.01 by two-tailed Student's *t*-test or one-way analysis of variance ANOVA. HCC, hepatocellular carcinoma; STX6, syntaxin-6.

To determine the biological effects of STX6 on the development of HCC, we first detected the expression of STX6 at the mRNA and protein levels in different HCC lines (Fig. S2A, B). We then selected MHCC-97H, Hep3B, and PLC/PRF/5 cells to establish stable STX6 knockdown cell lines and stable STX6-overexpressing Huh7, HCC-LY10, and Li-7 cell lines. The knockdown and overexpression efficiencies were validated by qRT-PCR and western blotting (Fig. 2A, B and Fig. S2C, D). Subsequently, we used the Cell Counting Kit 8 and 5-ethynyl-2-deoxyuridine (EdU) labeling assays to detect cell proliferation in stably STX6-deficient or STX6-overexpressing HCC cell lines. We found that STX6 overexpression promoted HCC cell proliferation whereas STX6 knockdown resulted in a marked reduction in proliferation (Fig. 2C, D and Fig. S2E, F). Similarly, an *in vitro* colony formation assay indicated that STX6 overexpression enhanced the clonogenicity of HCC cells whereas STX6 knockdown resulted in weaker clone-forming ability (Fig.2E-G and Fig. S2G-I). In addition, we found that STX6 knockdown resulted in a cell cycle G1/S arrest whereas STX6 overexpression resulted in a significant increase in the cell population in the S phase (Fig. 2H and Fig. S2J, K).

After investigating the role of STX6 in HCC *in vitro*, we implanted STX6-knockdown MHCC-97H cells in situ and subcutaneously transplanted STX6-overexpressing Huh7 cells into nude mice to observe the effect of STX6 on tumorigenicity *in vivo*. Consistent with our *in vitro* results, the tumor size and weight of nude mice overexpressing STX6 were significantly larger than those of the control group. Conversely, tumor size was significantly reduced in the STX6 knockdown group compared to that in the negative control group (Fig. 2I, J). We then verified the expression of STX6 in xenografted tumor tissues by qRT-PCR and western blot assays and found that STX6 expression in MHCC-97h cells was lower than that in the control group whereas that in Huh7 cells was higher (Fig. S2L-N). Collectively, these results showed that STX6 promoted HCC tumorigenicity and tumor tissue growth.

### STX6 promotes HCC metastasis in vitro and in vivo

**Figure 3:**
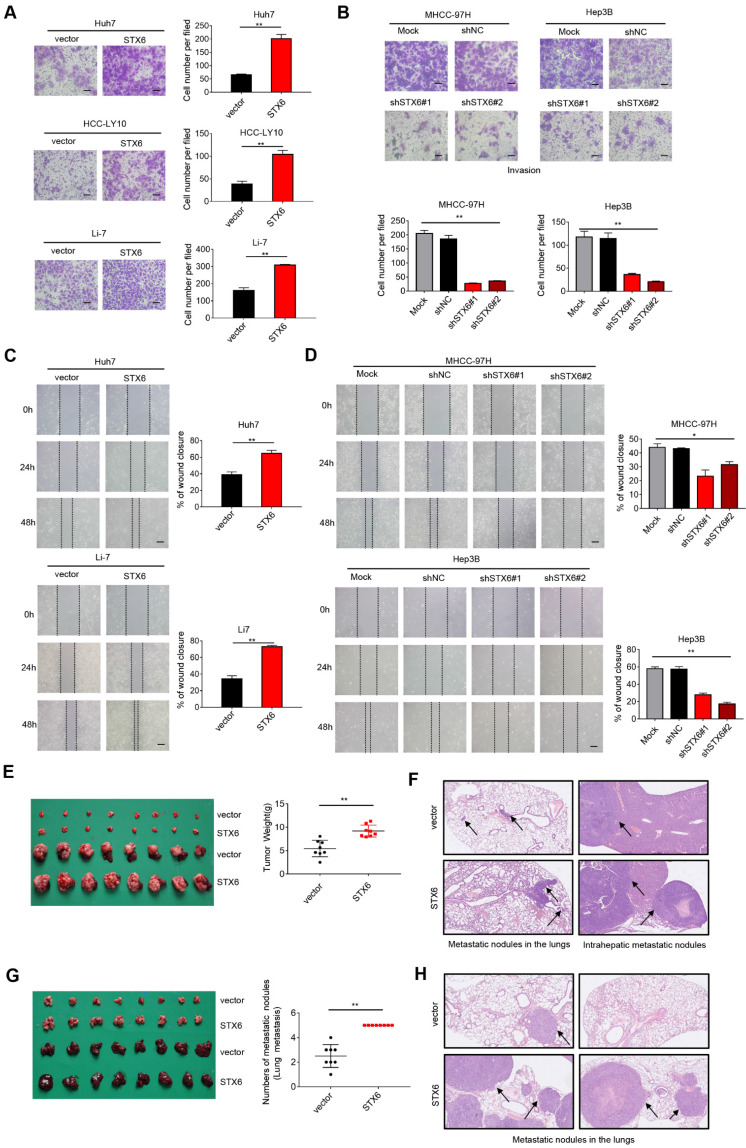
STX6 promotes HCC migration and invasion. (**A**) Transwell invasion assay of Huh7, HCC-LY10, and Li-7 cells with STX6 overexpression. Scale bar: 100 µm. (**B**) Representative images of Transwell assays for invasion of STX6-deficient MHCC-97H and Hep3B cells. Scale bar: 100 µm. (**C**) Scratch wound assay of STX6-overexpressing Huh7 and Li-7 cells. Scale bar: 50 µm. (**D**) Representative images of scratch wound assay for migration of STX6-deficient MHCC-97H and Hep3B cells. Scale bar: 50 µm. (**E**) In situ transplantation models of human-derived STX6 overexpression and control Li7 cells in the liver and quantitative statistical plots. (**F**) Representative images of metastatic nodes in the lungs as well as in the liver in an animal model of hepatic in situ tumor implantation. The black arrow indicates tumor nodules. (**G**) Animal model of tail vein injection of Li-7 cells and quantitative statistics of the number of lung lobe metastasis. (**H**) Representative images of lung metastatic nodules in 2 mice of STX6 overexpression and control group by tail vein injection. The black arrow indicates tumor nodules. Data for the *in vitro* experiments are the means ± SDs and are representative of three independent experiments. *p < 0.05, **p < 0.01 by two-tailed Student's *t*-test or one-way analysis of variance ANOVA. HCC, hepatocellular carcinoma; STX6, syntaxin-6.

To explore the effects of altering the expression of STX6 on the metastatic and invasive potential of HCC cells, we performed *in vitro* cell mobility (Transwell) experiments. The results showed that the invasiveness of HCC cells was enhanced after STX6 overexpression (Fig. 3A), whereas it was significantly reduced after STX6 knockdown compared to the control group (Fig. 3B and Fig. S3A, B). Furthermore, the results of the in vitro wound healing assays indicated that the overexpression of STX6 significantly promoted the migratory ability of HCC cells. Conversely, knockdown of STX6 could inhibit the migratory ability of HCC cells (Fig. 3C, D and Fig. S3C, D). Subsequently, we placed STX6 overexpressing HCC cells to hepatic in situ to establish an orthotopic liver tumor model and simultaneously performed tail vein injection experiments. The STX6 overexpression group had heavier orthotopic liver tumors (Fig. 3E) and larger lung and intrahepatic metastatic nodules (Fig. 3F), but the number of metastases was not statistically significant. And the tail vein injection model suggested that STX6 promoted the number and volume of pulmonary metastatic nodules in mice with HCC (Fig. 3G, H). Collectively, our findings indicated that STX6 markedly promoted HCC cell metastasis in vitro and in vivo.

### STX6 is downregulated by USF2 in HCC cells

Our results showed that STX6 is a positive regulator of HCC. Therefore, we aimed to determine the regulatory factors underlying its high expression in HCC cells. To predict the potential transcription factors of STX6, we collected Venn diagrams using the UCSC Genome Browser, JASPAR, ALGGEN, and htfTARGET databases (Fig. S4A). Among them, the upstream stimulatory factor (USF) 1/2 attracted our attention because it has been poorly studied in HCC. therefore, we focused on USF1 and USF2.

**Figure 4:**
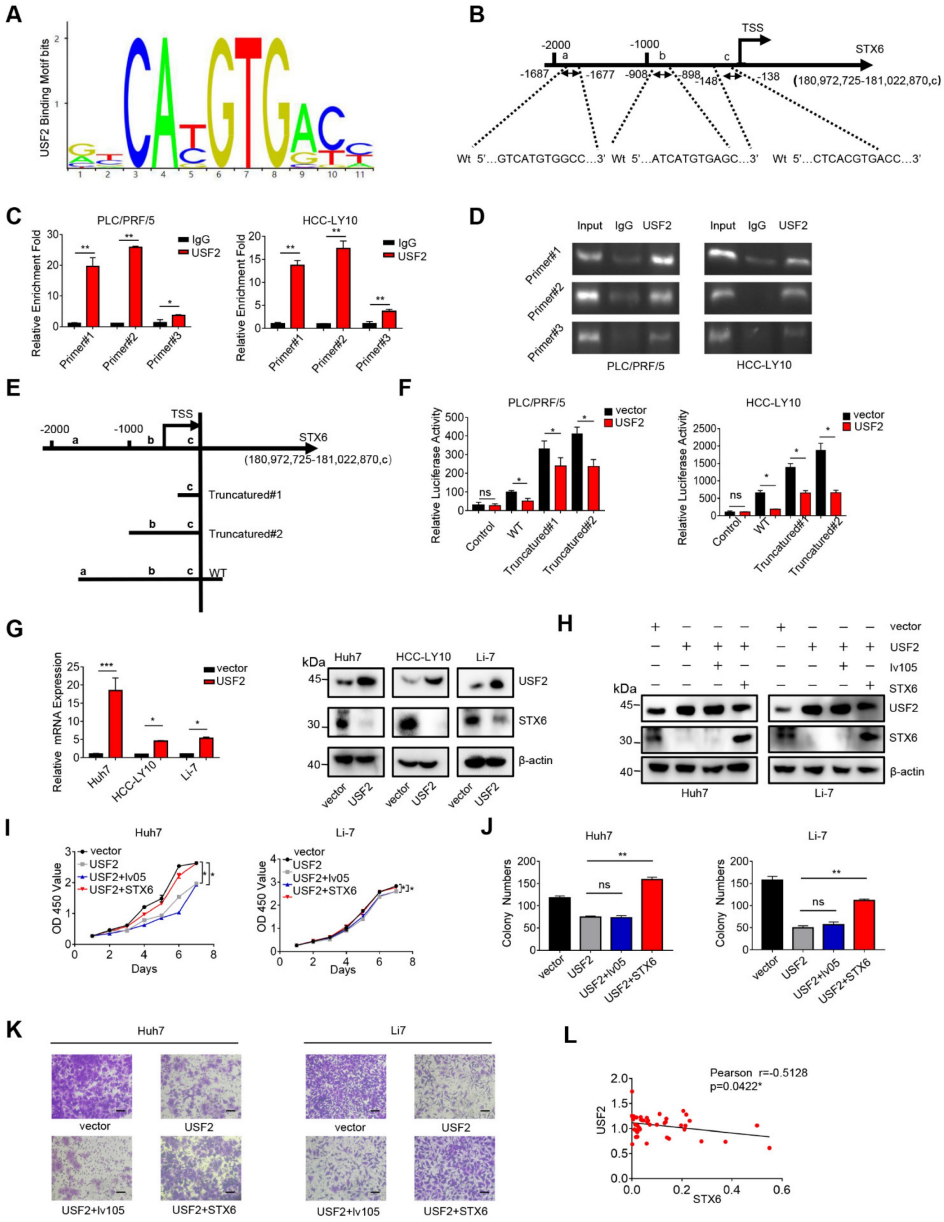
USF2 binds to the STX6 promoter and inhibits STX6 expression. (**A**) JASPAR analysis of USF2 potential binding sites. (**B**) JASPAR analysis showed three potential USF2-binding sites of the promoter region of STX6. (**C**) Chromatin immunoprecipitation (ChIP) quantitative real-time polymerase chain reaction (qPCR) analysis of USF2 binding to the STX6 promoter in PLC/PRF/5 and HCC-LY10 cells. (**D**) Agarose electrophoresis for ChIP analysis of USF2 binding to the STX6 promoter. (**E**) Luciferase reporter vectors containing truncation mutants of the promoter region of STX6 transfected into PLC/PRF/5 and HCC-LY10 cells. (**F**) Dual-luciferase reporter assays of the corresponding luciferase activities. (**G**) Western blot and q-PCR analysis of the protein and mRNA expression levels, respectively, of USF2 and STX6 in HCC cells after USF2 overexpression. (**H**) The protein levels of STX6 and USF2 in HCC cells co-overexpressing STX6 and USF2. (**I and J**) Cell Counting Kit-8 (**I**) and colony formation (**J**) assays of STX6 and USF2 co-overexpressing HCC cells. (**K**) Quantitative analysis of the Transwell assays of the invasion of HCC cells co-overexpressing STX6 and USF2. (**L**) STX6 mRNA level is negatively correlated with USF2 mRNA levels in 47 paired HCC tissues and adjacent matched noncancerous tissues. Data for the *in vitro* experiments represent the mean ± SD and are representative of three independent experiments. *p < 0.05, **p < 0.01 by two-tailed Student *t*-test or one-way analysis of variance ANOVA. HCC, hepatocellular carcinoma; STX6, syntaxin-6; USF2, upstream stimulatory factor 2, Pearson correlation coefficient.

We ruled out the possibility of USF1 binding to the STX6 promoter region using the chromatin immunoprecipitation (ChIP) assay (Fig. S4B). Next, we analyzed the possible USF2 binding sites on the STX6 promoter using the JASPAR database and predicted three possible binding regions (represented by a, b, and c, respectively) located at -1687~-1677 bp (a), -908~-898 (b), and -148~-138(c) relative to the transcription start site (TSS) (Fig. 4A, B). The ChIP assay also showed that USF2 was recruited to these three sites (Fig. 4C, D). Furthermore, we established three different dual-luciferase reporter plasmids, as shown in Fig. 4E: a 2213 bp STX6 promoter region and two truncated clones of the region (truncated#1: -363-200 bp relative to the TSS; truncated#2: -1122- 200 bp relative to the TSS). Dual-luciferase experiments revealed that after the STX6 promoter and the transcription factor USF2-binding region were truncated, STX6 exhibited stronger transcriptional activity, and all three binding regions had transcriptional repression effects in PLC/PRF/5 and HCC-LY10 cells (Fig. 4F). Moreover, we established stable HCC cells overexpressing USF2, and confirmed USF2 overexpression through qPCR. We then detected the expression level of STX6 using western blotting and found that it was downregulated (Fig. 4G). Therefore, our results indicated that USF2 was a transcriptional repressor of STX6.

### USF2 rescues the effects of STX6 overexpression in HCC

The HCCDB website showed that the expression of USF2 in HCC tissues was lower than that in normal paracancerous tissues (Fig. S5A). The canSAR Black website showed that the pathological grade of patients with HCC is negatively correlated with USF2 expression (Fig. S5B). Moreover, patients with HCC with upregulated USF2 expression on the kmplot website had a better prognosis (Fig. S5C). Therefore, to explore the function of USF2 in HCC, we stably overexpressed USF2 in HCC cells, and revealed that USF2 overexpression inhibited HCC cell proliferation, migration, and invasion (Fig. S5D-G). Furthermore, we overexpressed STX6 in USF2-overexpressing cells (Fig. 4H and S6A) and showed that the inhibition of cell proliferation, migration, and invasion induced by USF2 overexpression was rescued by STX6 overexpression (Fig. 4I-K and S6B-F). In addition, we analyzed the mRNA levels of USF2 and STX6 in tumor tissues collected from patients with liver cancer and found that USF2 and STX6 expression levels were negatively correlated (Fig. 4L). Collectively, these results indicated that USF2 inhibited cell proliferation and metastasis by suppressing STX6 expression in HCC.

### STX6 regulates autophagy in HCC cells

**Figure 5:**
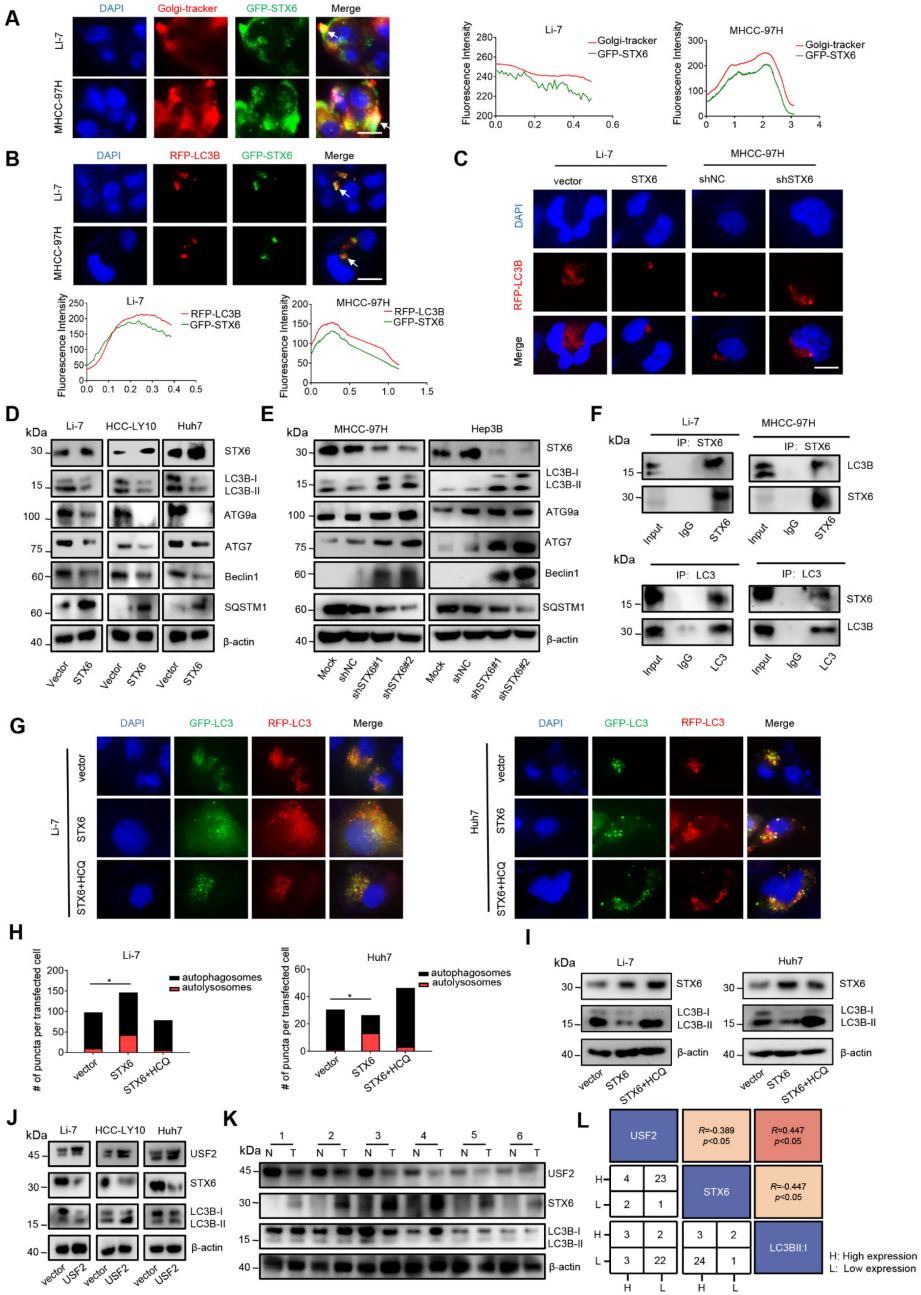
STX6 promotes autophagy flux in HCC cells. (**A**) Immunofluorescence assay of the colocalization of STX6 with the Golgi apparatus and its merged images. The graph shows the Golgi signal intensity measured by the red line in the MHCC-97H cell and Li7 cell image, and the green line indicates the signal observed of EmGFP-STX6. (**B**) Colocalization of RFP-LC3B and EmGFP-STX6 in MHCC-97H and Li-7 cells. The red line measures the autophagosome signal intensity and the green line represents the signal intensity of EmGFP-STX6. (**C**) Representative immunofluorescence images of autophagosomes in STX6 knockdown MHCC-97H cells and STX6-overexpressing Li7 cells. (**D**) Western blot of autophagy-related proteins in HCC cells after STX6 overexpression. (**E**) Western blot of autophagy-related proteins in HCC cells after STX6 knockdown. (**F**) Coimmunoprecipitation was STX6 and LC3A/B. The assay was performed using anti-LC3A/B and anti-STX6 antibodies. IgG served as a negative control. (**G**) Representative images of STX6-overexpressing Li-7 and Huh7 cells transiently transfected with tandem mCherry/GFP-tagged MAP1LC3B plasmid and co-cultured with hydroxychloroquine. (**H**) Quantification of the ratio of autophagosomes (yellow spots on overlay) and autolysosomes (red spots on overlay). Scale bar: 10 µm. (**I**) Western blot of LC3B expression in cells co-cultured with hydroxychloroquine after overexpression of STX6. (**J**) STX6 and LC3B expression in USF2-overexpressing HCC cells were determined using Western blot. (**K**) Western blotting analysis of USF2, STX6, and LC3B-II/I expression levels in HCC tissues (T) and paired noncancerous tissues (N). (**L**) Correlation analysis of USF2, STX6, and degree of autophagy. Error bars represent the mean ± SD, n=3. *, *p* < 0.05; **, *p* < 0.01 by two-tailed Student's *t*-test or one-way analysis of variance ANOVA. HCC, hepatocellular carcinoma; STX6, syntaxin-6; GFP: green fluorescent protein.

STX6 is mainly located in the trans-Golgi network^15^. Therefore, to further explore the molecular mechanism of STX6 in HCC, we used GFP-tagged STX6 to determine its localization, and found that STX6 partially overlapped with the Golgi apparatus; however, STX6 was also scattered in the cytoplasm (Fig.5A). Moreover, STX6 mediates the fusion of endophagosomes and autophagosomes and accelerates xenophagy by promoting autophagy^11^. To explore the role of STX6 in autophagic pathways in HCC, we investigated the localization of copGFP-fused STX6 and RFP-LC3B in Li-7 and MHCC-97H cells (Fig. 5B). Our results showed that STX6 was spatially coincident with autophagosomes. We then investigated the changes in the number of autophagosomes in HCC cells after STX6 overexpression and knockdown. We found that after 6 h of Earle's balanced salt solution (EBSS) culture, fewer autophagosomes were formed in STX6-overexpressing Li-7 cells than in their control group whereas significantly more autophagosomes were formed in STX6-deficient MHCC-97H cells (Fig. 5C). Next, we performed western blotting to detect changes in LC3B-I and LC3B-II expression levels after STX6 overexpression or knockdown. Our results showed that STX6 was able to regulate autophagy in HCC cells (Fig. S7A).

To further understand the effects of STX6 on autophagy, we evaluated the changes in the expression of key proteins in the autophagic pathway after STX6 knockdown or overexpression in HCC cells. The results showed that autophagy-related molecules decreased and the accumulation of SQSTM1 increased after STX6 overexpression, and this effect was reversed after STX6 knockdown (Fig. 5D, E). Similar results were observed in the mouse tumor xenografts (Fig. S7B, C). These results indicated that STX6 regulated autophagy in HCC.

### STX6 promotes LC3 degradation by accelerating the autophagic flux

Autophagy is generally divided into three steps: formation of autophagosomes, fusion of autophagosomes and lysosomes into autophagolysosomes, and degradation of autophagolysosomes^16^. To further explore the specific mechanism of the effect of STX6 on autophagy, we selected LC3, ATG7, ATG9a, P62, and Beclin1, which play key roles at different stages of autophagy and performed co-immunoprecipitation (Co-IP) experiments with STX6. The Co-IP analysis demonstrated that STX6 interacted with LC3B-I (Fig. 5F and S7D). LC3B-I is activated by conjugation to the amino group of the lipid phosphatidylethanolamine (PE) (LC3-I/PE) through the E1 (ATG7) and E2 (ATG3) enzymes. Moreover, LC3B-II is closely related to autophagosome formation^17^. Therefore, these results suggested that STX6 inhibited autophagy in HCC cells by downregulating LC3B expression.

To determine the reasons underlying the reduction in LC3 proteins, we transfected stable STX6-overexpressing and STX6-knockdown cells with tandem fluorescent mCherry/GFP-tagged MAP1LC3B plasmids to analyze the autophagic flux^18^. Because GFP is inactivated under acidic conditions, autophagosomes are shown in yellow in this assay, while autophagolysosomes are shown in red. The ratio of red to yellow dots was compared to analyze whether the autophagic flow was unobstructed. We observed that the ratio of autophagolysosomes (red dots) to autophagosomes (yellow dots) increased after STX6 overexpression. After adding hydroxychloroquine (HCQ) to block the fusion of autophagosomes and lysosomes, the ratio of yellow to red dots was reduced (Fig. 5G, H). Moreover, we detected changes in the protein expression of LC3B-I and LC3B-II in LI-7 and HUH7 cells overexpressing STX6 after adding HCQ and found that HCQ attenuated the effect of STX6 on LC3B-II (Fig. 5I). This indicates that STX6 overexpression promoted the association of autophagosomes with lysosomes and accelerated the degradation of LC3.

To further explore the correlation of USF2, STX6, and degree of autophagy with clinical prognostic values, we detected the expression levels of these proteins using western blotting in USF2 overexpressing HCC cells and clinical samples of 36 patients with HCC. The results showed that USF2 could regulate autophagy in HCC cells by regulating the expression of STX6(Fig. 5J). And as shown in Fig. 5K and Fig. S7E. We analyzed the data of HCC clinical samples and matched noncancerous liver tissues and found that the degree of autophagy flux increased as STX6 expression levels increased. Moreover, quantitative analysis showed that USF2 and STX6 were negatively correlated in HCC. and both were statistically significant (Fig. 5L). These results indicated that STX6 promoted autophagic flux in HCC cells.

### STX6 overexpression increases the sensitivity of HCC cells to rapamycin

**Figure 6:**
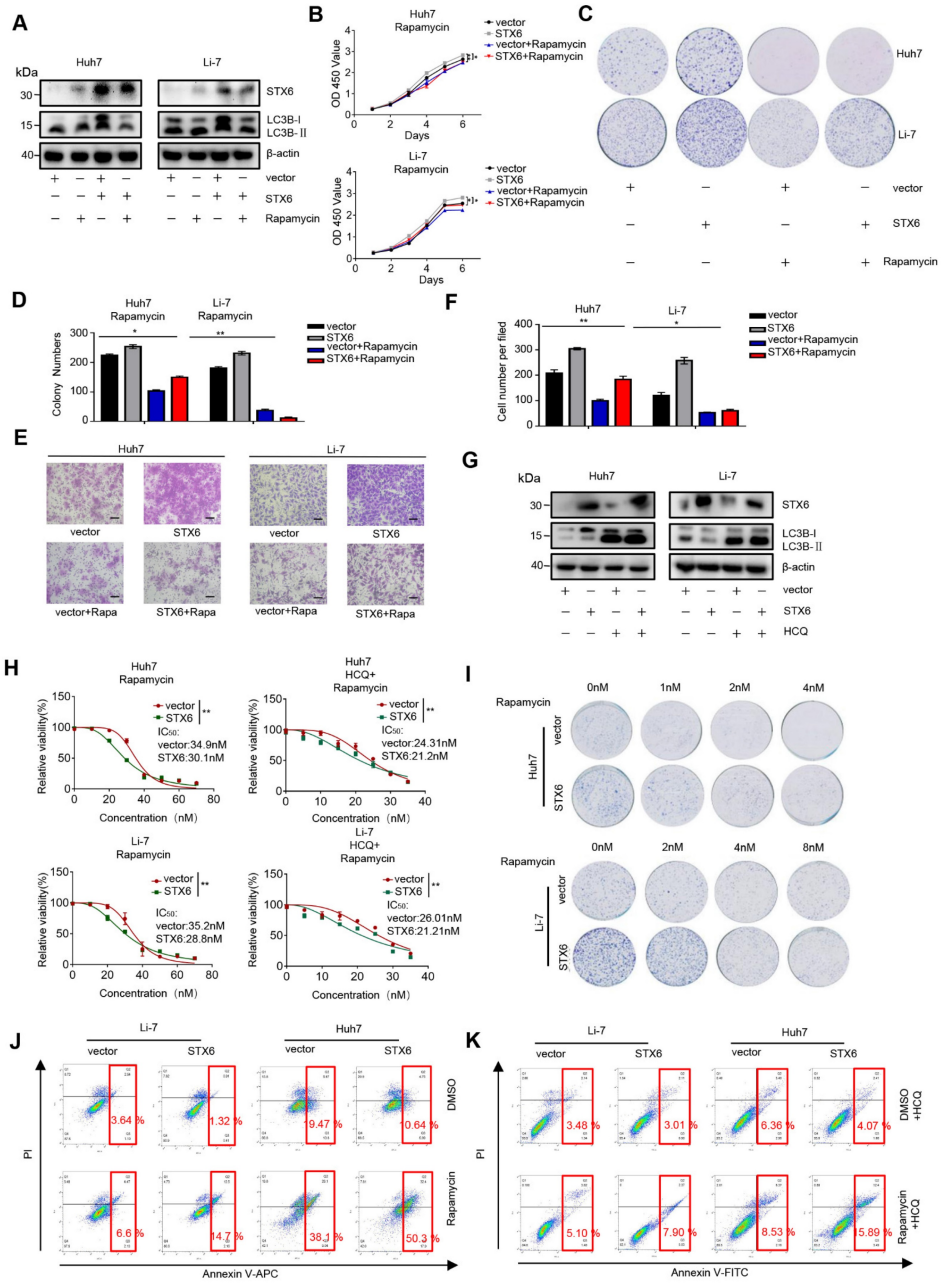
STX6-overexpression sensitizes HCC cells to rapamycin by regulating autophagy. (**A**) Western blot analysis of STX6 and autophagy-related proteins in STX6-overexpressing HCC cells treated with rapamycin. (**B**) Cell Counting Kit-8 assay of the proliferation of STX6-overexpressing HCC treated with rapamycin. (**C-D**) Colony formation of STX6-overexpressing HCC cells treated with rapamycin. Bar graphs show the quantitative analysis of colony numbers. (**E**) Transwell invasion assay of STX6-overexpressing HCC cells treated with rapamycin. (**F**) Quantitative data analysis of Transwell assays to quantify the invasion of STX6-overexpressing HCC cells. (**G**) Expression of STX6 and LC3II/I in STX6 overexpressing HCC cells treated with rapamycin and HCQ. (**H**) Cell viability of STX6-overexpressing Li-7 and Huh7 cells treated with various concentrations of rapamycin (with or without 20 μM HCQ) for 72 h. (**I**) Long-term colony formation assays of STX6-overexpressing Huh7 and Li-7 cells treated with rapamycin. (**J**) Flow cytometric analysis of apoptosis of the apoptotic rate in STX6-overexpressing Li-7 and Huh7 treated with rapamycin. (**K**) Flow cytometric analysis of the apoptotic rate in STX6-overexpressing Li-7 and Huh7 treated with rapamycin and HCQ. Error bars represent the mean ± SD, n=3. * *p* < 0.05; ** *p* < 0.01 by two-tailed Student's *t*-test or one-way analysis of variance ANOVA. HCC, hepatocellular carcinoma; STX6, syntaxin-6.

Rapamycin targets mTOR1 to activate cellular autophagy, and mTOR1 can inhibit the ULK complex by phosphorylating complex components^19^. Therefore, we investigated whether STX6 overexpression could alter the sensitivity of HCC cells to rapamycin. First, we detected the degree of autophagy after treating STX6-overexpressing HCC cells with rapamycin and detected the expression levels of STX6 and LC3 expression levels using western blotting. We observed that the change of autophagy following STX6 overexpression in HCC cells was rescued by treatment with rapamycin (Fig. 6A). Furthermore, rapamycin rescued the enhanced proliferative capacity of HCC cells after STX6 overexpression (Fig. 6B-D) and reduced the metastatic and invasive ability of STX6-overexpressing cells (Fig. 6E, F). Subsequently, we investigated the changes in the sensitivity of HCC cells to rapamycin after STX6 overexpression and explored whether these alterations were associated with autophagy. We examined changes in LC3B protein by Western blot assay in HCC cells overexpressing STX6 versus control groups after the addition of HCQ (Fig. 6G). The viability of STX6 overexpressing HCC cells was determined after 72h of culture in a medium with increasing concentrations of rapamycin (with or without HCQ), and dose-dependent toxicity curves and IC_50_ values were calculated. As shown in Fig. 6H, STX6 overexpression reduced the IC_50_ of HCC cells for rapamycin. And the difference of IC_50_ between overexpressing STX6 and control groups could be reduced by the addition of HCQ. We then investigated the effects of STX6 overexpression on the long-term resistance of HCC cells to rapamycin using clonogenic assays.

The results showed that rapamycin cytotoxicity was increased in HCC cells overexpressing STX6 (Fig. 6I and Fig. S8A). In addition, using flow cytometry, we detected the apoptosis level of STX6-overexpressing and control cells after adding rapamycin and found that the apoptotic rate in the STX6 overexpression group was higher than that in the control group (Fig. 6J and Fig S8B). Consistently, the difference in the proportion of apoptotic cells between the overexpressing-STX6 group and controls became smaller after the addition of HCQ (Fig. 6K and Fig S8C). Collectively, these results indicated that STX6 overexpression enhanced the sensitivity of HCC cells to rapamycin by promoting autophagy flux.

### STX6 enhances the resistance of HCC cells to therapeutic drugs

**Figure 7:**
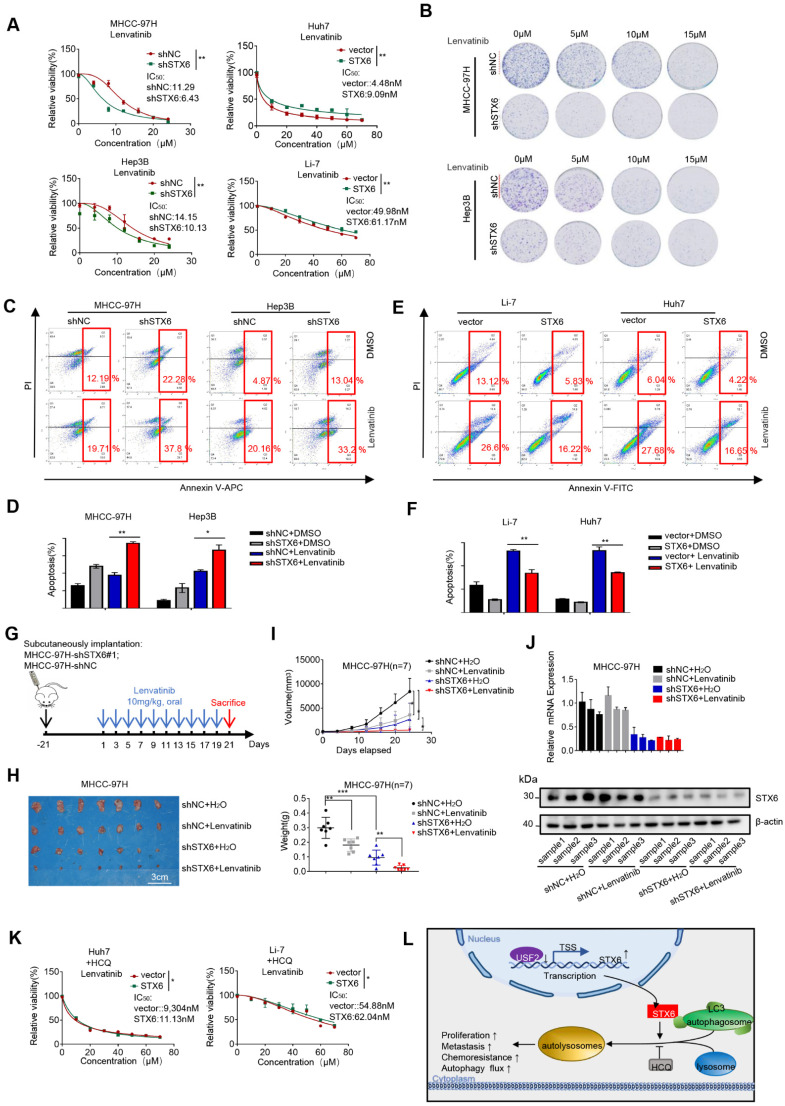
STX6 promotes HCC resistance to lenvatinib. (**A**) Cell viability of STX6 overexpressing or knockdown HCC cells treated with different concentrations of lenvatinib for 72h. (**B**) Long-term colony formation assay of STX6-deficient MHCC-97H and Hep3B cells treated with lenvatinib. (**C-D**) Flow cytometric analysis of STX6-deficient MHCC-97H and Hep3B cells treated with lenvatinib and its quantitative analysis. (**E-F**) Flow cytometric analysis of the apoptotic rate of STX6 overexpressing HCC cells treated with lenvatinib and its quantitative analysis. (**G**) MHCC-97H -shSTX6/MHC-LM3-shNC cells were subcutaneously injected into nude mice. After 21 days of tumor formation, the mice were treated with 10 mg/kg lenvatinib orally every day. (**H**) Treatment with lenvatinib and STX6 knockdown inhibited tumor growth, and STX6 knockdown combined with lenvatinib treatment effectively abolished tumor growth in mice (n=7). (**I**) STX6 protein expression levels in liver tissues of mice bearing xenografts from MHCC-97H cells with stable STX6 knockdown and lenvatinib treatment group. (**J**) Quantitative data analysis of tumor weight and volume. (**K**) Cell viability of STX6-overexpressing HCC cells treated with different concentrations of lenvatinib and 20 μM HCQ for 72 h. (**L**) Pattern diagram illustrating the tumor-promoting role of the USF2-STX6-LC3B axis in HCC. Error bars represent the mean ± SD, n=3. * *p* < 0.05; ** *p* < 0.01 by a two-tailed Student's *t*-test or one-way analysis of variance ANOVA. HCC, hepatocellular carcinoma; STX6, syntaxin-6.

Lipid rafts/cholesterol-enriched membrane microdomain CEMM deficiency results in the release of VAMP3 and STX6, which promotes succulent doxorubicin resistance in breast cancer^20^. To study the effect of STX6 on the response to clinical treatments of HCC, we investigated the effect of STX6 overexpression or knockdown on the sensitivity of HCC cells to lenvatinib. First, we determined the cell viability curves and IC_50_ changes in STX6-overexpressing or STX6-deficient cells co-cultured with lenvatinib for 72h. STX6-overexpressing HCC cells were more resistant to lenvatinib and STX6-deficient HCC cells were more sensitive to lenvatinib (Fig. 7A). Subsequent long-term toxicity clone plate test results were consistent with the above results (Fig. 7B and Fig.S8D). Flow cytometric analysis revealed that the apoptotic ratio of STX6-deficient cells was higher than that of the control group after treatment with lenvatinib (Fig. 7C, D). However, the apoptosis ratio was reduced in STX6 overexpressing cells compared with control cells (Fig. 7E, F).

To confirm these *in vitro* results, we subcutaneously injected STX6-deficient HCC cells and their control cells into nude mice and then treated them with distilled water or lenvatinib by gavage for two weeks (Fig. 7G). The results are shown in Fig. 7H-I. STX6 knockdown enhanced the antitumoral effects of lenvatinib in HCC. We then determined the protein and mRNA expression levels of STX6 in the mouse tumors and confirmed that STX6 knockdown was successful (Fig. 7J). Therefore, STX6 knockdown enhanced the sensitivity of HCC cells to lenvatinib. And the difference between IC_50_ to levatinib between overexpressing STX6 and the control group could be reduced by the addition of HCQ (Fig. 7K).

Collectively, our results indicated that USF2 inhibited the expression of STX6 and that STX6 promoted autophagic flux and affected the sensitivity of HCC cells to chemotherapeutic drugs (Fig. 7L).

## Discussion

In our study, we found that the expression of STX6 was upregulated in HCC tissues and was closely related to the prognosis of patients with HCC. Our results showed that STX6 overexpression promoted the proliferation, migration, and invasion of HCC cells. Mechanistically, STX6 accelerated LC3 degradation by accelerating the autophagic flux in HCC cells. Moreover, USF2 can downregulate the expression of STX6, and USF2 overexpression rescued the effects of STX6 on HCC cells. Interestingly, we also found that HCC cells with upregulated STX6 expression had enhanced drug resistance but were more sensitive to rapamycin. Our study is the first to reveal the clinical implications of STX6 expression in HCC.

The SNARE family of proteins is involved in intracellular membrane fusion events. SNARE proteins have an unusual ability to form a trans-complex, thereby stabilizing the transition state and facilitating liposome fusion^21^. The expression of STX6 has been reported to be high in several tumors. A previous study has suggested that Vps13B selectively associates with recycling endosomes carrying STX6 and operates as a tethering factor in the transport from early endosomes to recycling endosomes^22^. In addition, circSTX6 can promote pancreatic ductal adenocarcinoma proliferation and metastasis 23. STX6 can also act as a prognostic biomarker for patients with papillary renal cell carcinoma, and STX6 inhibitors are a promising novel therapy against renal cell carcinoma^24^. The roles of STX6 vary in different tumors, however, there are few related reports on STX6 in HCC. In our study, we revealed that STX6 was upregulated in HCC tissues and promoted the progression of HCC cells.

Upstream transcription factor 2 (USF2) is involved in the regulation of multiple cellular processes. More than half of USF2-deficient mice die quickly, suggesting that USF2 is necessary for survival^25^, ^26^. The transcriptional regulation of USF2 plays a role in the development of various human tumors. S100A8, a calcium- and zinc-binding protein, is regulated by the TGF-β/USF2 axis and induces epithelial‐mesenchymal in colorectal cancer 27.

In addition, hsa-miR-875-5p inhibits tumorigenesis and TGF-β signaling in gastric cancer by targeting USF2^28^. Moreover, studies in an *in vivo* mouse xenograft model revealed that the overexpression of USF2 in prostate cancer cells reduces tumorigenicity^29^, ^30^. USF2 deficiency induces reactive oxygen species formation and contributes to the activation of the ERK1/2-AKT pathway, thereby promoting tumor proliferation and migration^31^. In general, conflicting data on the role of USF2 in tumor development suggest that it acts as both a tumor promoter and suppressor. However, little is known about the role of USF2 in the occurrence of HCC. In the present study, we found that USF2 inhibits HCC progression by directly repressing STX6 expression.

Autophagy is a mechanism that transports cellular material to lysosomes for degradation, thereby providing cells with energy and macromolecular precursors. Autophagy has opposite roles in different cancers, and interventions that stimulate or inhibit autophagy can be used as cancer therapies^32^. Disruption of the Beclin1/VPS34/Atg14 complex in an Akt-dependent manner negatively regulates autophagy to stabilize HIF-1α and promote HCC proliferation 33. This indicates that the occurrence and development of HCC may be related to autophagy regulation. In our study, we found that STX6 overexpression promoted autophagic flux in HCC cells, thereby increasing their sensitivity to rapamycin. Multiple preclinical and clinical data suggest that rapalogs are effective in enhancing antitumor activity in conjunction with targeted therapies^34^. In this study, we found that the degree of resistance to lenvatinib increased after STX6 overexpression which was in contrast to the result obtained with rapamycin. Therefore, our experiments provide a new approach to the clinical treatment of HCC using a combination of rapamycin.

Collectively, our study demonstrated for the first time the tumor-promoting role of STX6 in HCC and confirmed that high STX6 expression promoted HCC proliferation and metastasis in vivo and vitro and promoted autophagic flux in HCC cells. We also verified that USF2 inhibited HCC growth by negatively regulating the transcription of STX6. Our findings provide the first evidence of a USF2/STX6/LC3B regulatory axis in HCC, which may be a potential target for the treatment of HCC.

## Supplementary Material

Supplementary figures and tables.

## Abbreviations

CCK8: Cell Counting Kit 8
EBSS: Earle's Balanced Salt Solution
GFP: green fluorescent protein
HCC: hepatocellular carcinoma
MAP1LC3B/LC3B: microtubule-associated protein 1 light chain 3 beta
IHC: immunochemistry
mRNA: messenger RNA
SNARE: soluble N-ethylmaleimide-sensitive factor attachment protein receptor
STX6: syntaxin-6
TCGA: The Cancer Genome Atlas
USF2: upstream stimulatory factor 2
