# Supplementary material for: Syntaxin-6 promotes the progression of hepatocellular carcinoma and alters its sensitivity to chemotherapies by activating the USF2/LC3B axis
Source: Int J Biol Sci. 2023 Jul 31;19(12):3892–907. doi: 10.7150/ijbs.86636 (PMC10411479; doi:10.7150/ijbs.86636)
Supplement: Supplementary file 1 — Supplementary figures and tables. [file ijbsv19p3892s1.pdf]

Syntaxin-6 promotes the progression of hepatocellular carcinoma and alters its sensitivity to chemotherapies by activating the USF2/LC3B axis

Lianer Zhou[1], Zhenyu Wang[2], Xiaoxia Chen[3], Xianxian Li[1], Chao Ge[1], Xuejie Min[1], Fangyu Zhao[1], Taoyang Chen[4], Jinjun Li[1*]

[1] State Key Laboratory of Systems Medicine for Cancer, Renji Hospital, Shanghai Jiao Tong University School of Medicine, Shanghai 200032, China

[2] Department of Oncology, Shanghai General Hospital, Shanghai Jiaotong University School of Medicine, Shanghai 200080, China

[3] School of Life Science and Technology, Shanghai Tech University, Shanghai 201210, China

[4] Qi Dong Liver Cancer Institute, Qi Dong 226200, China

***Correspondence:** jjli@shsci.org;

Supplementary Materials

Supplementary Tables

Table S1. The sequences of shRNA Target

Identifier	Target sequence (5'-3')
shSTX6#1	GAACTGGAGCACTGGAACAAC
shSTX6#2	GCAGCAGTTGATCGTGGAACA

Table S2. The sequences of gene-specific primers used for qRT-PCR and ChIP assay

Gene name	Forward(5'-3')	Reverse(5'-3')
	Primers for qRT-PCR	
STX6	AGCAGGCACAGCAGCAGTT	AGGATGAGCACAACCAACAGGA
USF2	GCGTTCGGCGACCACAACAT	GGACTGCCACCATTGCTGAAGG
	Primers for ChIP	
ChIP-1	TCTCTGTTCTCAATCGCTGTCA	AACCTCAAGATCCCAAAGGACT
ChIP-2	AAATGGATGGTGTGGTGGCTCA	TTTCCTAGGCTGGCCTTGAACA
ChIP-3	TTGAGGAGAGGGCTGTAGAAGC	AATCACGAACGCCGCCTGAA

Table S3. Antibodies used in this study.

Antibody	Catalog	Dilution	Company	Detection
STX6	PA5-30366	1:500	Invitrogen	WB/IHC
USF2	sc-293443	1:200	Santa Cruz	WB/ChIP
β-actin	A3854	1:10000	Sigma	WB
Atg7	8558S	1:1000	CST	WB/CO-IP
Atg9A	13509S	1:1000	CST	WB/CO-IP
SQSTM1/p62	5114S	1:1000	CST	WB/CO-IP
Beclin-1	3495T	1:1000	CST	WB/CO-IP
MAP1LC3A/B	12741S	1:1000	CST	WB
HRP conjugated goat anti-rabbit IgG	A0545	1:5000	Sigma-Aldrich	WB
HRP conjugated goat anti-mouse IgG	A0168	1:5000	Sigma-Aldrich	WB

Table S4. Correlation between STX6 expression levels in HCC patients and their clinicopathologic characteristics.

Clinical pathology	STX6 expression			P value
	Negative (%)	Low (%)	High (%)	
Gender				
male	33(84.62)	58(73.42)	81(83.51)	0.182
female	6(15.38)	21(26.58)	16(16.49)	
Age				
≤50	29(74.36)	52(65.82)	62(64.58)	0.535
＞50	10(25.64)	27(34.18)	34(35.42)	
AFP（ng/ml)				
≤20	12(30.77)	28((36.84)	34(35.42)	0.808
＞20	27(69.23)	48(63.16)	62(64.58)	
HBV infection				
absent	6(16.67)	15(19.48)	16(16.84)	0.887
present	30(83.33)	62(80.52)	79(83.16)	
Tumor size (cm)				
≤5	17(44.74)	42(54.55)	45(46.88)	0.449
＞5	21(55.26)	35(45.45)	51(53.12)	
Histological grade				
I-II	16(41.03)	31(39.24)	59(60.82)	0.009*
III-IV	23(58.97)	48(60.76)	38(39.18)	
Intrahepatic metastasis				
absent	26(66.67)	51(26.58)	70(72.16)	0.541
present	13(33.33)	28(73.42)	27(27.84)	
Cirrhosis				
absent	6(15.38)	12(15.19)	15(15.46)	0.999
present	33(84.62)	67(84.81)	82(84.54)	

Supplementary Figures

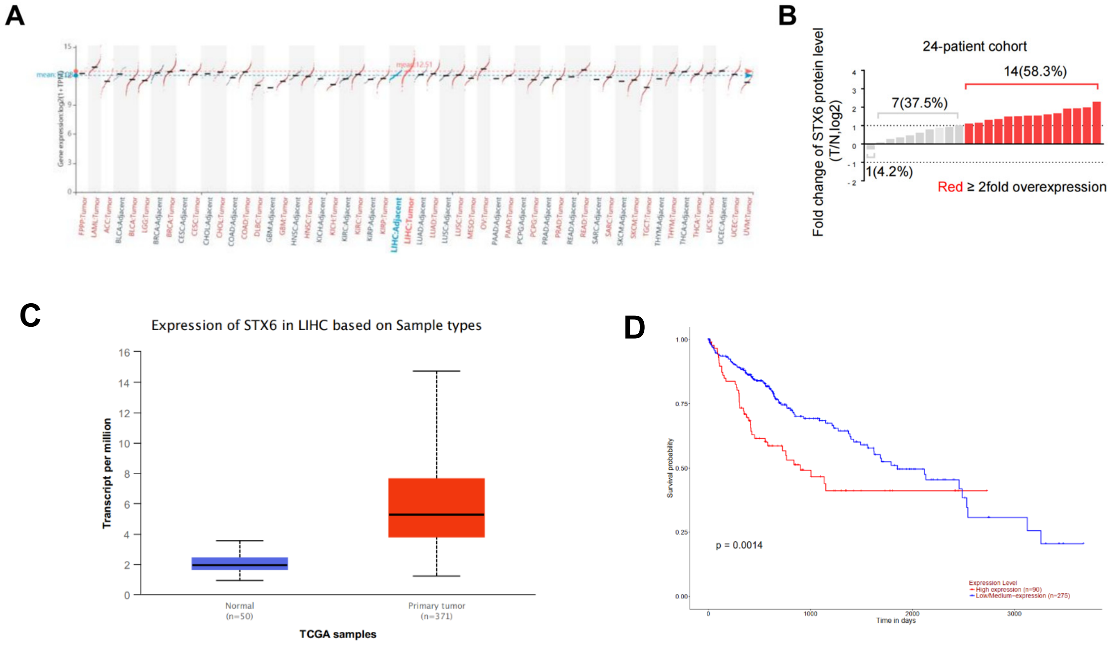

Figure S1. STX6 is a potential biomarker in HCC. **A** The expression of STX6 in different cancers of TCGA dataset was analyzed by the HCCDB database (http://lifeome.net/database/hccdb/home.html). **B** Waterfall plot showing the protein level of STX6 in HCC compared with adjacent noncancerous tissues from 24 patients, determined by Western blot assays. Red histogram, STX6 overexpressed more than two times. **C** Differential expression of STX6 in tumor and paraneoplastic tissues of HCC in the UALCAN database. **D** Prognostic correlation analysis between STX6 expression level and HCC patients in the UALCAN database.

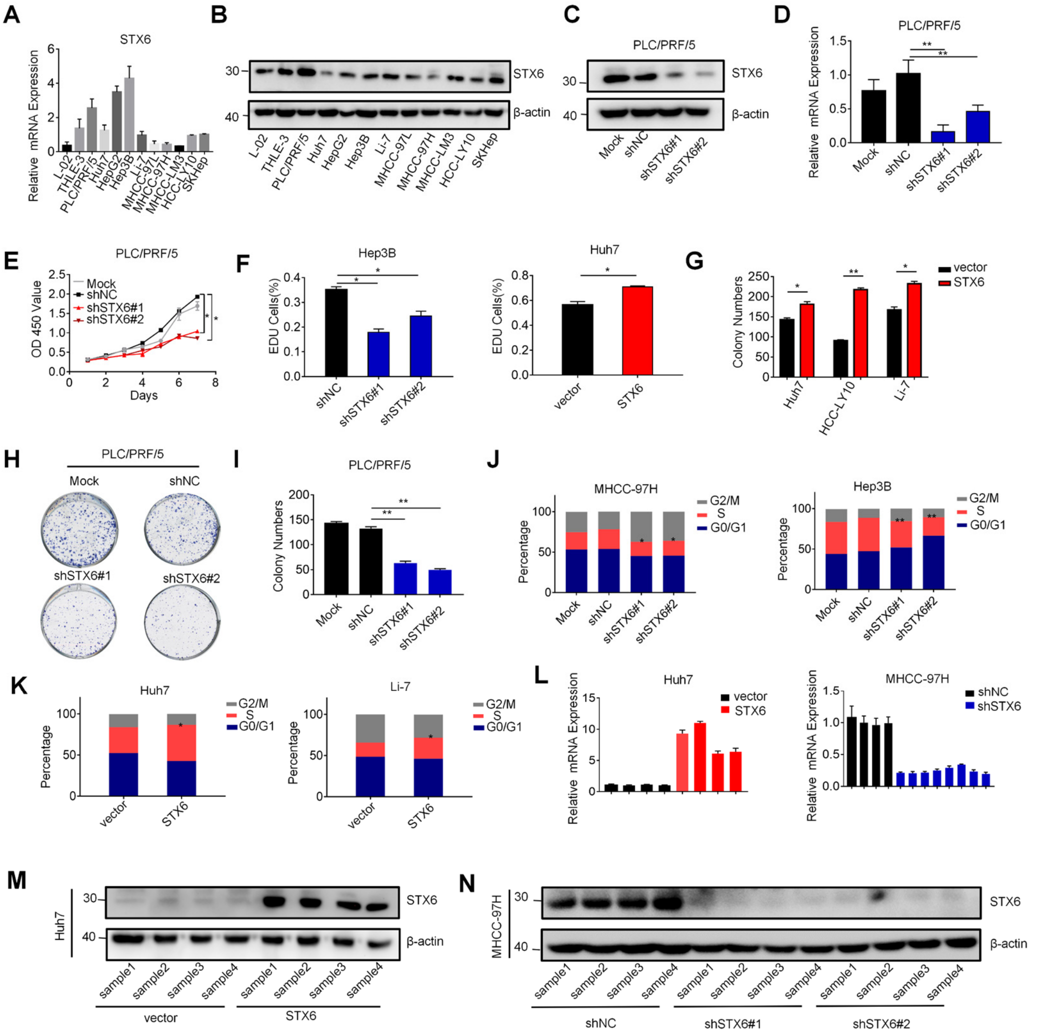

Figure S2. STX6 promotes proliferation and cell cycle of HCC cells. **A** The mRNA expression level of STX6 in human HCC cell lines. **B** Protein expression levels of STX6 in human HCC cell lines. **C-D** Protein and mRNA levels of STX6 in PLC/PRF/5 cells after STX6 knockdown. **E**The cell proliferation ability of STX6 knockdown in PLC/PRF/5 cells was assessed by the CCK8 assay. **F** Quantitative data of the EdU labeling assays of STX6 overexpressed or knockdown HCC cells. **G** The cell proliferation was evaluated by colony formation assay in STX6 overexpressed HCC cells. **H-I** Colony formation assay and quantitative data showing the proliferation of the STX6-knockdowning PLC/PRF/5 cells. **J-K** Flow cytometry used

to analyze the cell cycle status of HCC cells with STX6 overexpression(J) and cells with silencing STX6 (K). DNA content was quantified using Modfit 3.2 software. **L** STX6 mRNA levels were detected in liver tissues from animals bearing xenografts from Huh7 and MHCC-97H. **M-N** STX6 protein expression levels were detected in liver tissues from animals bearing xenografts from Huh7 and MHCC-97H cells with stable STX6 overexpression and knockdown. Data are the mean of biological triplicates and are shown as the mean ± SD. p values: $^{*}p < 0.05$, $^{**}p < 0.01$; by two-tailed Student t test.

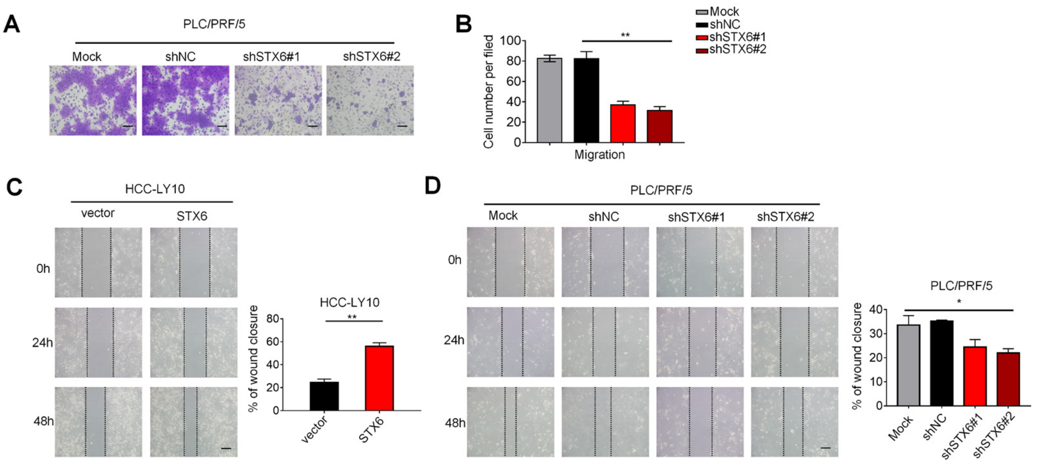

Figure S3. STX6 promotes invasion and migration ability in HCC cells. **A** The cell invasion effects of STX6 knockdown PLC/PRF/5 cell were assessed by *in vitro* transwell invasion assay. **B** Quantitative data of transwell assays for invasion of STX6 knockdown PLC/PRF/5 cell. Scale bar: 100 μm. **C** Scratch wound assay of STX6-overexpressing HCC-LY10 cells. Scale bar: 50 μm. **D** Representative images of scratch wound assay for migration of STX6-deficient PLC/PRF-5cells. Data are the mean of biological triplicates and are shown as the mean ± SD. p values: *$p < 0.05$, **$p < 0.01$; by two-tailed Student t test.

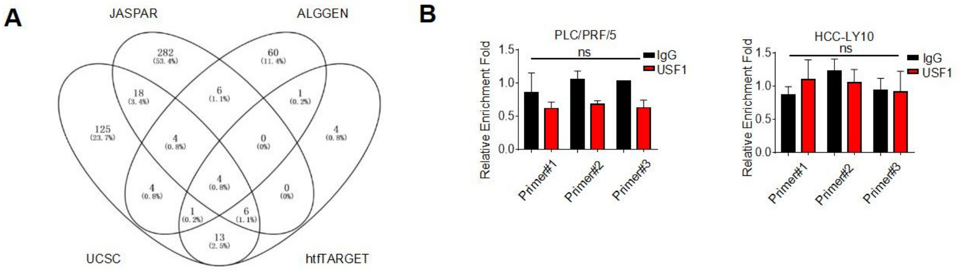

Figure S4. Prediction of upstream transcription factors of STX6. **A** Venn diagram of predicted STX6 upstream transcription factors using UCSC Genome Browser, JASPAR, ALGGEN and htfTARGET databases. **B** ChIP-qPCR analysis of USF1 binding to the STX6 promoter in PLC/PRF/5 and HCC-LY10 cells. Data are the mean of biological triplicates and are shown as the mean ± SD. p values: *$p < 0.05$, **$p < 0.01$; by two-tailed Student t test.

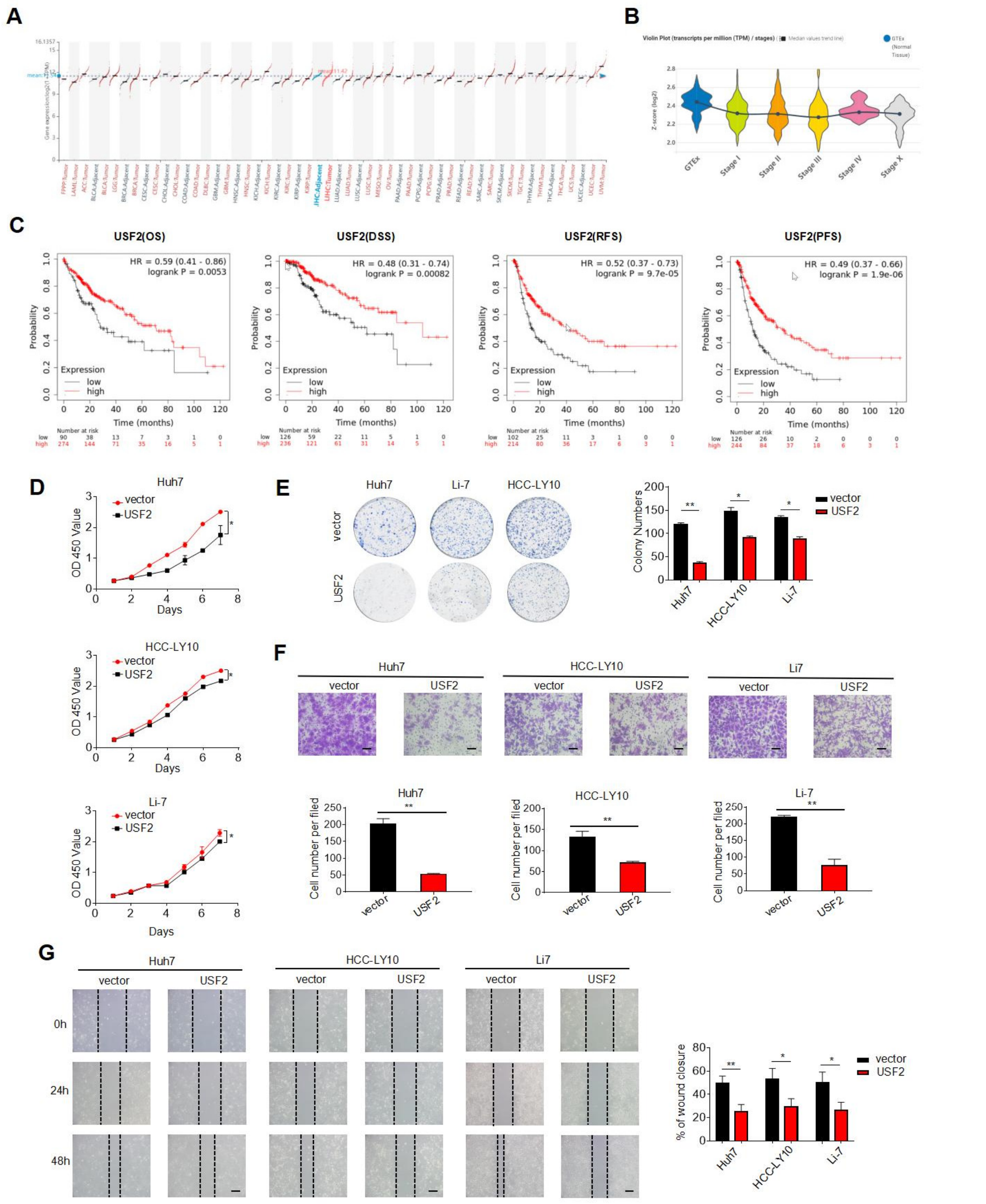

Figure S5. The expression and clinical significance of USF2 in HCC. **A** The expression of USF2 in different cancers of TCGA dataset was analyzed by the HCCDB database (http://lifeome.net/database/hccdb/home.html). **B** USF2 expression in HCC patients with different pathological stages analyzed from canSAR Black | the Cancer Drug Discovery Platform (icr.ac.uk). **C** The correlation between the mRNA

expression of USF2 and the survival of HCC patients in TCGA dataset was assessed from Kaplan-Meier plotter [Liver RNAseq] (kmplot.com). **D** HCC cell proliferation ability of USF2 overexpression cell lines were detected by CCK8 assay. **E** Representative images and quantitative data of colony formation assay showing the proliferation ability of the USF2 overexpressed HCC cells. **F** Representative images of the transwell assays of the USF2 function in HCC cell invasion. Histogram graphs show the quantitative data. Scale bar: 100 μm. **G** Cell migration of USF2 overexpressing cell lines was assessed using a scratch wound assay. Scale bar: 50 μm. Data are the mean of biological triplicates and are shown as the mean ± SD. p values: *p < 0.05, **p < 0.01; by two-tailed Student t test.

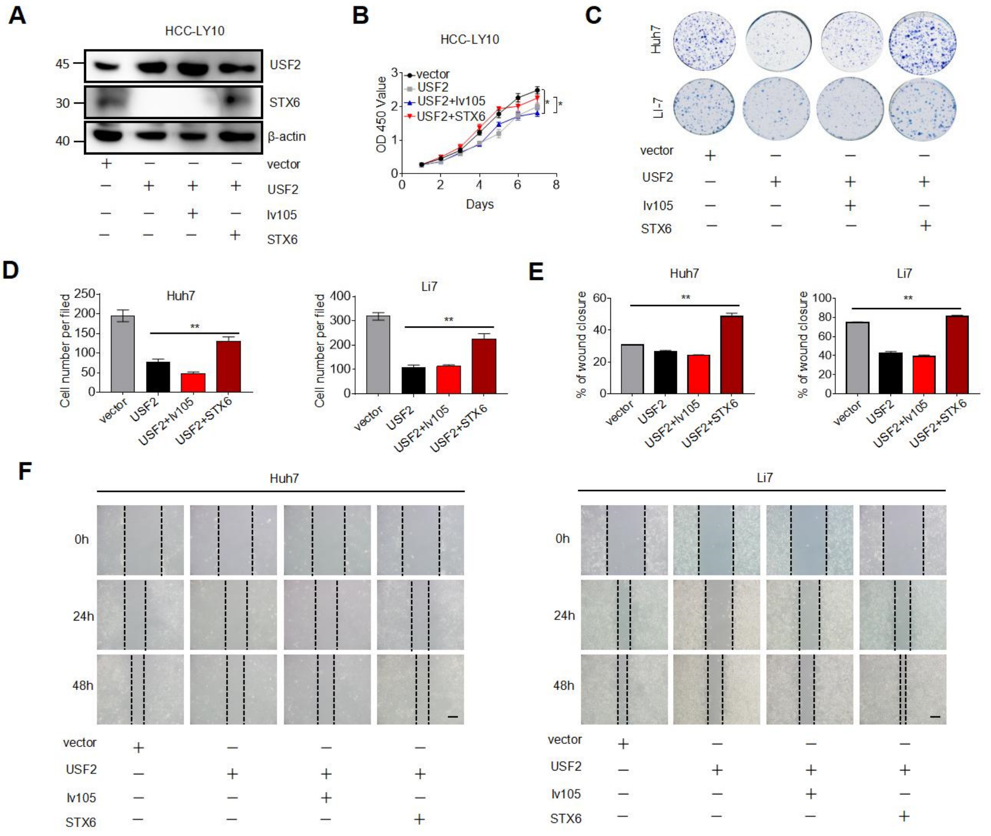

Figure S6. USF2 inhibits STX6 to inhibit HCC progression **A** The protein levels of STX6 and USF2 in STX6 as well as USF2 co-overexpressing in HCC-LY10 cells. **B-C** The cell proliferation of STX6 and USF2 co-overexpressing was evaluated by CCK8 and colony formation assays in HCC-LY10 cells. **D** Evaluation of migration and invasion abilities of cells co-overexpressed with STX6 and USF2 using transwell assay. **E-F** Cell migration ability of cell lines with USF2 and STX6 co-overexpression were assessed using an in vitro scratch assay. Scale bar: 50 μm. Data are the mean of biological triplicates and are shown as the mean ± SD. p values: *p < 0.05, **p < 0.01; by two-tailed Student t test.

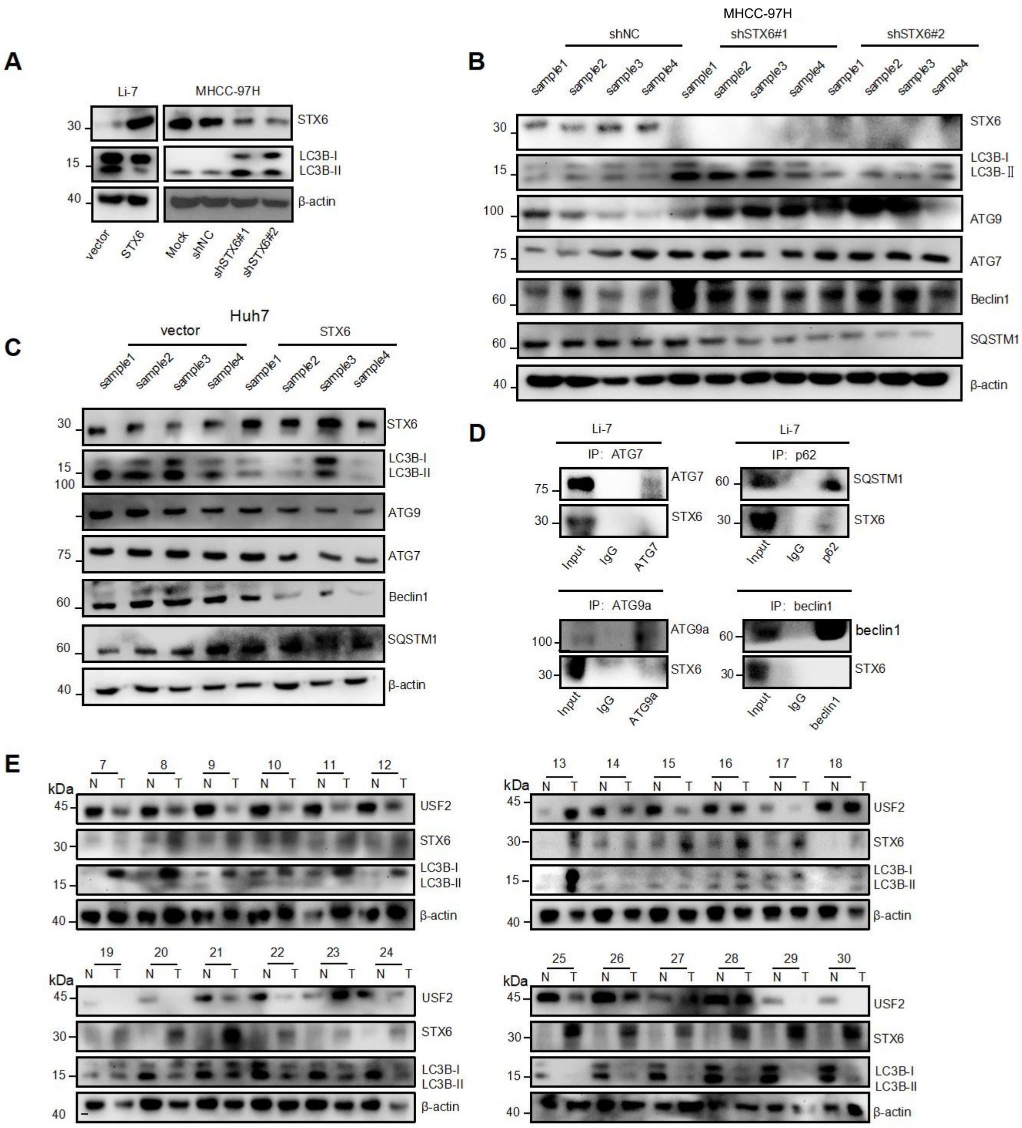

Figure S7. STX6 regulates autophagy pathway in HCC. **A** Protein expression of LC3B after STX6 overexpression or knockdown. **B-C** The protein level of STX6, LC3B and autophagy pathway in liver tumors from animals with tumor xenografts inoculated with MHCC-97H and Huh7 cell lines after STX6 overexpression and knockdown. **D** CO-IP of autophagy pathway proteins in HCC cells in Li-7 cell. **E** STX6 protein level was negtively correlated with the USF2 and negatively correlated

with the LC3B-II/I levels in 30 paired HCC tissues and the adjacent matched noncancerous tissues.

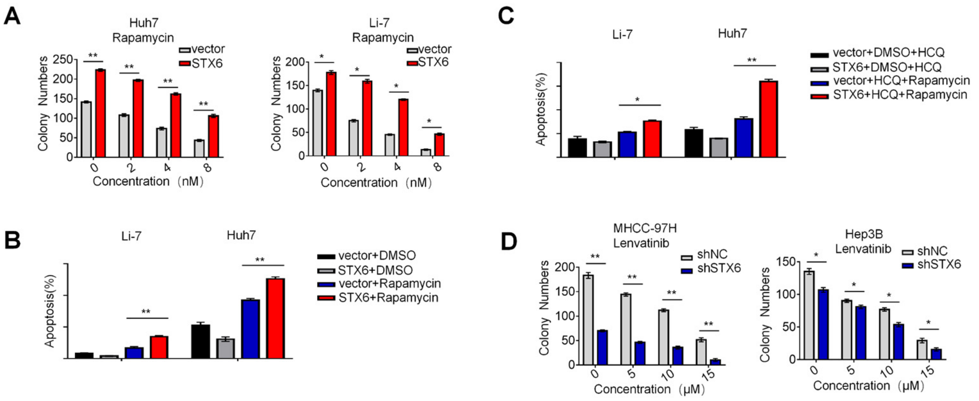

Figure S8. STX6 changes the chemosensitivity of HCC by regulating autophagy. **A** The bar chart shows the quantitative analysis of rapamycin long-term drug toxicity test. **B** Quantitative analysis of the colony formation assay for the long-term drug toxicity test of levatinib. **C-D** Quantitative analysis of apoptotic flow cytometry.
